# Discovery of Phenolic Glycoside from *Hyssopus cuspidatus* Attenuates LPS-Induced Inflammatory Responses by Inhibition of iNOS and COX-2 Expression through Suppression of NF-κB Activation

**DOI:** 10.3390/ijms222212128

**Published:** 2021-11-09

**Authors:** Xingyu Liu, Jie Su, Geng Wang, Lihua Zheng, Guannan Wang, Ying Sun, Yongli Bao, Shuyue Wang, Yanxin Huang

**Affiliations:** 1 National Engineering Laboratory for Druggable Gene and Protein Screening, Northeast Normal University, Changchun 130117, China; liuxy013@nenu.edu.cn (X.L.); suj445@nenu.edu.cn (J.S.); wanggn258@nenu.edu.cn (G.W.); suny040@nenu.edu.cn (Y.S.); baoyl800@nenu.edu.cn (Y.B.); 2 NMPA Key Laboratory for Quality Control of Cell and Gene Therapy Medicine Products, Northeast Normal University, Changchun 130117, China; wangg666@nenu.edu.cn (G.W.); zhenglh015@nenu.edu.cn (L.Z.)

**Keywords:** anti-inflammation, *Hyssopus cuspidatus*, hyssopuside, macrophages, NF-κB, phenolic glycoside

## Abstract

It seems quite necessary to obtain effective substances from natural products against inflammatory response (IR) as there are presently clinical problems regarding accompanying side effects and lowered quality of life. This work aimed to investigate the abilities of hyssopuside (HY), a novel phenolic glycoside isolated from *Hyssopus cuspidatus* (*H. cuspidatus*), against IR in lipopolysaccharide (LPS)-induced RAW 264.7 cells and mouse peritoneal macrophages. The results indicated that HY could reduce nitric oxide (NO) production and inhibit the production and secretion of pro-inflammatory mediators including tumor necrosis factor-α (TNF-α), interleukin-6 (IL-6), and interleukin-1β (IL-1β) in LPS-stimulated macrophages. Moreover, data from the immunofluorescence study showed that HY suppressed nuclear translocation of nuclear factor-kappa B (NF-κB) upon LPS induction. The Western blot results suggested that HY reversed the LPS-induced degradation of IκB (inhibitor of NF-κB), which is normally required for the activation of NF-κB. Meanwhile, the overexpression of inducible nitric oxide synthase (iNOS) and cyclooxygenase-2 (COX-2) diminished significantly with the presence of HY in response to LPS stimulation. On the other hand, HY had a negligible impact on the activation of mitogen-activated protein kinase (MAPK) pathways. Moreover, an in silico study of HY against four essential proteins/enzymes revealed that COX-2 was the most efficient enzyme for the interaction, and binding of residues Phe179, Asn351, and Ser424 with HY played crucial roles in the observed activity. The structure analysis indicated the typical characterizations with phenylethanoid glycoside contributed to the anti-inflammatory effects of HY. These results indicated that HY manipulated its anti-inflammatory effects mainly through blocking the NF-κB signal transduction pathways. Collectively, we believe that HY could be a potential alternative phenolic agent for alleviating excessive inflammation in many inflammation-associated diseases.

## 1. Introduction

Inflammation is an intricacy disease caused by the tissue injury or infection, along with the activation of diverse immune cells including macrophages, neutrophils, and lymphocytes [1]. The human diseases that are associated with these conditions, including obesity, type 2 diabetes, atherosclerosis, asthma, and neurodegenerative disease, are all characterized by chronic low-grade inflammation. Furthermore, the chronic para-inflammation that persists in these conditions, in turn, accelerates ulteriorly disease progression [2,3]. Recent evidence has suggested many chronic inflammatory diseases that are caused by infection or injury seem to be associated with conditions, including the continuous availability of high-calorie nutrients, a low level of physical activity, and old age [4]. Macrophages play a crucial role in secreting various inflammatory mediators such as nitric oxide (NO) and cytokines to host survival from infection and recover from tissue damage in basal conditions. Thus, the tissues are sustained in a homeostatic state [5]. In case of noxious conditions, inducers of inflammation such as lipopolysaccharide (LPS) can provoke the activity of immune cells and trigger the production of numerous inflammatory mediators, which in turn upset the functionality of many tissues and organs as well as the downstream effectors of the inflammatory pathway [6]. Hence, the control of inflammatory cytokines and mediators may have therapeutic potential in the treatment of various inflammatory diseases.

Nuclear factor-kappa B (NF-κB) exists in the cytoplasm in a quiescent manner combined with IκB (inhibitor of NF-κB) proteins, its inhibitory protein [7]. The pivotal regulatory event in NF-κB activation is the phosphorylation of the IκB kinase (IKK) complex, which leads to IκB protein ubiquitylation and subsequent degradation. Concomitantly, the activated NF-κB translocates to the nucleus where it activates the expression of target genes, including interleukin-6 (IL-6), interleukin-1β (IL-1β), and tumor necrosis factor-α (TNF-α); enzymes in the prostaglandin synthesis pathway (such as cyclooxygenase-2 (COX-2)); and inducible nitric oxide synthase (iNOS) [8,9,10]. Rising evidence has verified that the activation of NF-κB has definite pertinence with elevation of inflammatory cytokine levels in disease. Even with continuous research on mechanisms, current therapeutic approaches for chronic inflammatory disease have not been fully effective [11]. The accompanying severe side effects are even more unacceptable. Therefore, for the purpose of inhibiting abnormal activation of inflammatory pathways, this is a new strategy using non-toxic natural products as carriers to effectively control the course of chronic inflammation to prevent and resist inflammation-linked diseases.

*Hyssopus cuspidatus* (*H. cuspidatus*) is an edible and medicinal plant, widely distributed in the eastern Mediterranean, Central Asia, and parts of southern Europe, which has received rising attention and has excellent physiological bioactivity, including anti-inflammatory, anti-oxidation, anti-bacterial, anti-diabetic, and anti-asthmatic effects [12,13,14]. The ethanol extract from *H. cuspidatus* suppresses the production and expression of inflammatory cytokines and mediators, and further promotes the balance of Th1/Th2 immune regulation in ovalbumin induced bronchial asthma [13]. However, although a previous study focused on the activity of crude extract, few reports have regarded the correlations of the individually purified compound with anti-inflammatory activities, and the underlying anti-inflammatory mechanism has remained unclear.

**Figure 1:**
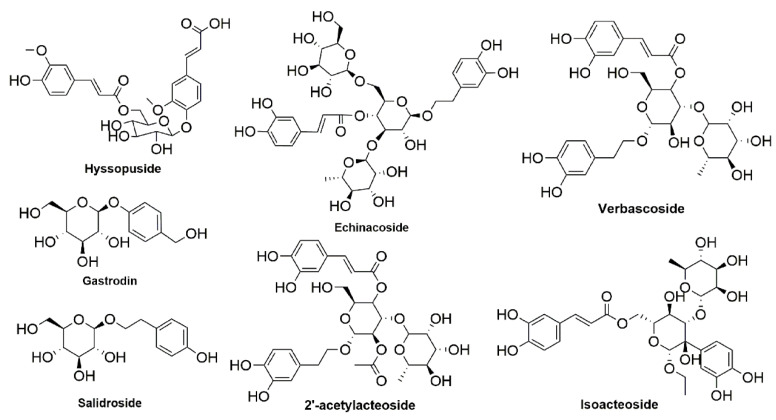
Chemical structures of phenolic glycoside with anti-inflammatory activity and hyssopuside.

To discover and verify plant-derived anti-inflammatory compounds is an important task in the field of phytochemistry. Naturally originated active products with multiple targets and little toxic side effects may have good potential in terms of drug likeness. In our previous research, a novel phenolic compound, hyssopuside (HY), was isolated and identified from *H. cuspidatus*, which exhibited significant anti-inflammatory activity [15]. HY possesses similar phenolic glycoside in structure compared with the natural anti-inflammatory drugs mentioned above (Figure 1 and Figure S1). The aim of this study was to investigate the role of HY in antagonizing LPS-stimulating RAW 264.7 cells and mouse peritoneal macrophages and to dissect the underlying mechanisms. Together, our results emphasize the importance of HY on anti-inflammation effects and provide evidence in applying effective drugs of *H. cuspidatus* for the prevention and treatment of inflammation-linked disorders.

## 2. Results

### 2.1. Effects of HY on the Viability of Macrophages

**Figure 2:**
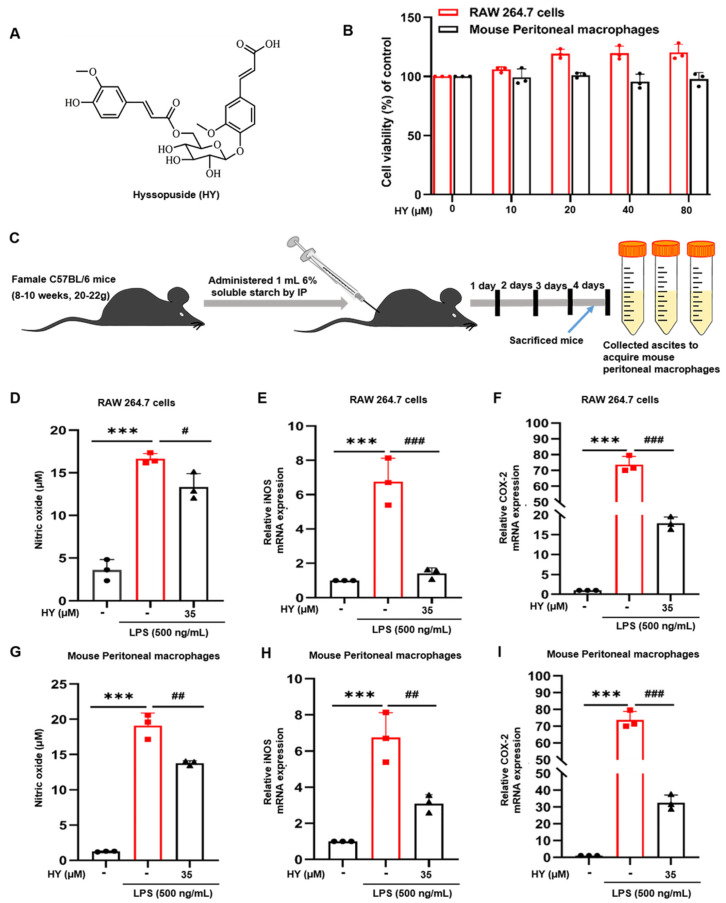
(**A**) Chemical structure of hyssopuside (HY). (**B**) Effects of HY on cell viability in RAW 264.7 cells and mouse peritoneal macrophages. Macrophages were treated with different concentrations of HY (10, 20, 40, and 80 μM) for 24 h. Cell viability was quantified with 3-(4,5-dimethylthiazol-2-yl)-2,5-diphenyltetrazolium bromide (MTT) assay. (**C**) Scheme of collecting ascites to acquire mouse peritoneal macrophages. The blue arrow means that mice were sacrificed on the fourth day after injecting intraperitoneally (IP) with 1 mL 6% soluble starch for three consecutive days. (**D**,**G**) The inhibitory effect of HY on lipopolysaccharide (LPS)-induced nitric oxide (NO) production in the supernatants of RAW 264.7 cells and mouse peritoneal macrophage. Following pre-treatment with HY (35 μM) for 3 h, macrophages were co-incubated with or without 500 ng/mL LPS for 24 h. (**E**,**H**) Real-time quantitative polymerase chain reaction (RT-qPCR) for the expression of inducible nitric oxide synthase (iNOS) on LPS-stimulated RAW 264.7 cells and mouse peritoneal macrophages. (**F**,**I**) RT-qPCR for the expression of cyclooxygenase-2 (COX-2) on LPS-stimulated RAW 264.7 cells and mouse peritoneal macrophages. Following pre-treatment with HY (35 μM) for 3 h, macrophages were co-incubated with or without 500 ng/mL LPS for 12 h. The total RNA was prepared, and the mRNA expression of COX-2 and iNOS were determined by RT-qPCR. Data are representatives of three replicates and shown as mean ± SD. *** *p* < 0.001 versus the control group; # *p* < 0.05, ## *p* < 0.01, ### *p* < 0.001 versus the LPS-treated group.

To determine the effects of HY (Figure 2A) on cell viability, we treated RAW 264.7 cells and mouse peritoneal macrophages with HY at different concentrations ranging from 0 to 80 µM for 24 h. The results of the MTT (3-(4,5-dimethylthiazol-2-yl)-2,5-diphenyltetrazolium bromide) assay (Figure 2B) indicated that HY showed no apparent cytotoxicity to RAW 264.7 cells. HY at 40 µM showed a slight cytotoxic effect to mouse peritoneal macrophages but had no statistic difference. Mouse peritoneal macrophages with higher concentrations of HY (80 µM) showed effects on cell viability similar to those treated with HY at 40 µM. To determine anti-inflammatory effects of HY on both two source macrophages, we sought to test the activity of HY at 35 µM throughout all experiments in this study.

### 2.2. Effects of HY on Inflammatory Mediator Production in LPS-Induced Macrophages

RAW 264.7 cells and mouse peritoneal macrophages are macrophages that play a critical role in the regulation of inflammatory disease. We investigated whether HY has the ability to regulate the inflammatory mediators of macrophages in response to LPS stimulation. We employed Griess regents to determine the level of NO in the supernatants collected from cells and utilized RT-qPCR assay to test the expression levels of pro-inflammatory mediators iNOS and COX-2 in the RNA extract form cells treated with 35 µM HY and stimulated with LPS for 12 h. The results showed that HY treatment potently inhibited LPS-induced production of NO (Figure 2D,G), as well as expression of iNOS (Figure 2E,H) and COX-2 (Figure 2F,I) in macrophages. These results suggested that HY could be able to suppress the production and secretion of inflammatory mediators from RAW 264.7 cells and mouse peritoneal macrophages stimulated with LPS.

### 2.3. Effects of HY on Inflammatory Cytokine Expression and Production in LPS-Induced Macrophages

**Figure 3:**
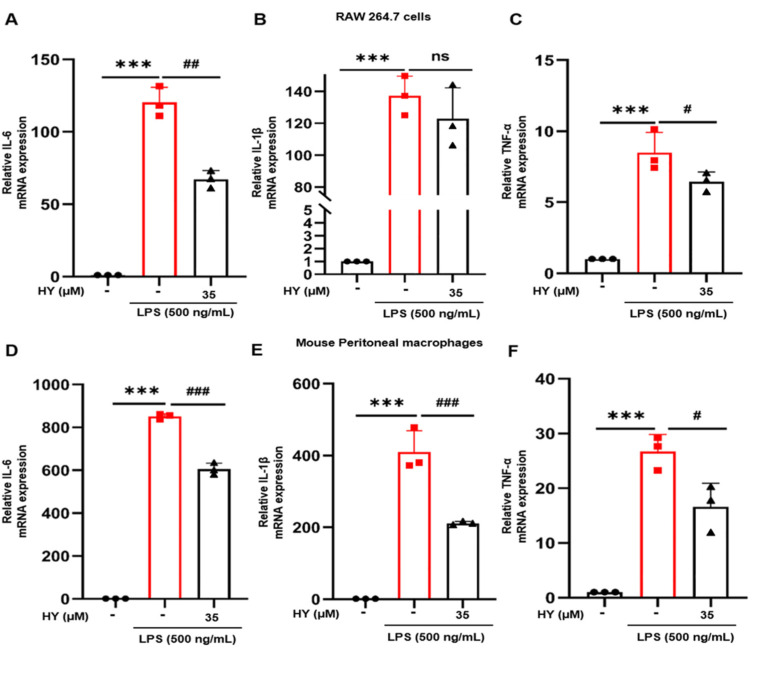
(**A**–**C**) Real-time quantitative polymerase chain reaction (RT-qPCR) for the expression of interleukin-6 (IL-6), interleukin-1β (IL-1β), and tumor necrosis factor-α (TNF-α) on lipopolysaccharide (LPS)-stimulated RAW 264.7 cells. (**D**–**F**) RT-qPCR for the expression of IL-6, IL-1β, and TNF-α on LPS-stimulated mouse peritoneal macrophages. Following pre-treatment with 35 μM hyssopuside (HY) for 1 h, RAW264.7 cells and mouse peritoneal macrophages were treated with or without 500 ng/mL LPS for 12 h. The total RNA was prepared, and the mRNA expression of IL-6, IL-1, and TNF-α were detected by RT-qPCR. Data are representatives of three replicates and shown as mean ± SD. *** *p* < 0.001 versus the control group; # *p* < 0.05, ## *p* < 0.01, ### *p* < 0.001 versus the LPS-treated group; ns: no significance versus the LPS-treated group.

Inflammatory cytokines involved in the inflammatory response include IL-6, IL-1β, and TNF-α, which can activate immune cells and cause inflammatory response. To further determine whether HY could inhibit the expression of inflammatory cytokines in LPS-stimulated RAW 264.7 cells and mouse peritoneal macrophages, we measured the expression levels of pro-inflammatory cytokines of IL-6, IL-1β, and TNF-α in the RNA extract form cells treated with 35 µM HY and stimulated with LPS for 12 h. The data suggested that HY could be able to suppress the expression of IL-6 and TNF-α from RAW 264.7 cells induced by LPS with statistical significance (Figure 3A,C). However, the effect of HY on the level of IL-1β had moderate inhibitory with no statistical significance difference in RAW 264.7 cells (Figure 3B). Similar with the results in RAW 264.7 cells, HY exhibited obviously inhibitory action on the expression of IL-6, IL-1β, and TNF-α in mouse peritoneal macrophages (Figure 3D–F).

**Figure 4:**
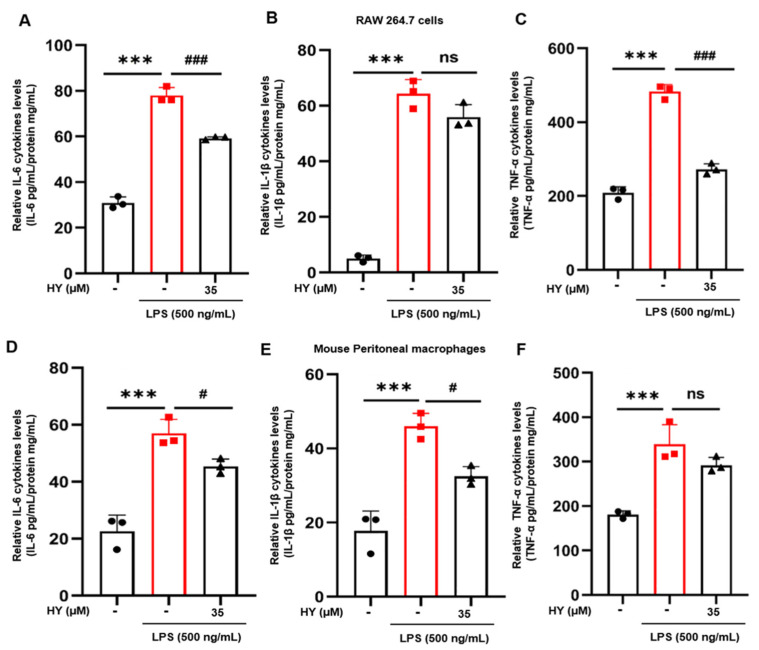
(**A**–**C**) Enzyme-linked immune sorbent assay (ELISA) for the secreted level of interleukin-6 (IL-6), interleukin-1β (IL-1β), and tumor necrosis factor-α (TNF-α) in the supernatants of cells treated with HY (35 μM) with 500 ng/mL lipopolysaccharide (LPS) for 24 h in RAW 264.7 cells. (**D**–**F**) ELISA for the secreted level of IL-6, IL-1β, and TNF-α in the supernatants of cells treated with 35 μM hyssopuside (HY) with 500 ng/mL LPS for 24 h in mouse peritoneal macrophages. Data are representative of three replicates and shown as mean ± SD. *** *p* < 0.05 versus the control group; # *p* < 0.05, ### *p* < 0.001 versus the LPS-treated group; ns: no significance versus the LPS-treated group.

Moreover, we applied the ELISA technique to measure the level of IL-6, IL-1β, and TNF-α in the supernatants collected from cells treated with 35 µM HY and stimulated with LPS for 24 h. The results indicated that HY treatment potently inhibited LPS-induced production of IL-6, IL-1β, and TNF-α in the supernatant of cells administrated with HY compared to the LPS group in RAW 264.7 cells (Figure 4A–C) and peritoneal macrophages of mice (Figure 4D,E). These data indicated that HY effectively reversed LPS-induced inflammatory response.

### 2.4. Effects of HY on Suppressing NF-κB Activation in LPS-Induced Macrophages

**Figure 5:**
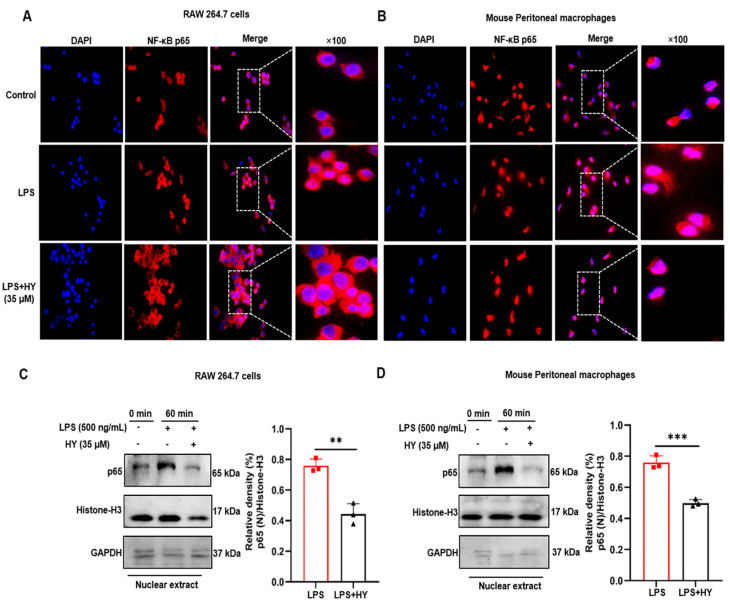
(**A**) The inhibitory effect of hyssopuside (HY) on nuclear localization of nuclear factor-kappa B (NF-κB) p65 in RAW 267.4 cells. (**B**) The inhibitory effects of HY on nuclear localization of NF-κB p65 in mouse peritoneal macrophages. Representative images from immunofluorescence assay showing NF-κB staining (red) in untreated cells, lipopolysaccharide (LPS)-stimulated cells, and HY-treated cells stimulated with LPS. Nuclei (blue) were stained with 4′,6-diamidino-2-phenylindole (DAPI). The micrographs were captured at 100× magnification, and data are representative of three replicates. (**C**) Western blot analysis for the inhibitory effect of HY on the expression of p65 in RAW 264.7 cells treated with 35 μM HY with 500 ng/mL LPS for 1 h and quantitative of p65. (**D**) Western blot analysis for the inhibitory effect of HY on the expression of p65 in mouse peritoneal macrophages treated with 35 μM HY with 500 ng/mL LPS for 1 h and quantitative of p65. Nuclear and cytosolic extracts were isolated, and the levels of p65 in each fraction were determined by Western blotting. Histone-H3 was detected as a nuclear internal control, and glyceraldehyde 3-phosphate dehydrogenase (GAPDH) was used as a cytosolic internal control. The histogram shows relative density ratios (fold changes) measured by ImageJ. Data are representatives of three replicates and shown as mean ± SD. ** *p* < 0.01, *** *p* < 0.001 versus the LPS group.

On the basis of our observation from RT-qPCR and ELISA that HY could potently suppress the production and secretion of NO, iNOS, COX-2, IL-6, IL-1β, and TNF-α in the RAW 246.7 cells and peritoneal macrophages stimulated with LPS, it is reasonable to assume that HY may suppress the activation of NF-κB in response to LPS stimulation. We therefore tested whether HY inhibited the LPS-induced translocation of NF-κB from the cytoplasm to the nucleus. We performed immunofluorescence assay to monitor the localization status of the p65 subunit of NF-κB. The results (Figure 5A,B) showed that macrophages without any treatment retained NF-κB in the cytoplasm of the cells, and the cells responded to LPS stimulation clearly revealed that NF-κB translocated into the nucleus where it normally functions to activate several different genes responsible for the synthesis of inflammatory mediators, such as iNOS, COX-2, and inflammatory cytokines, including IL-6, IL-1β, and TNF-α. Unquestionably, these data indicated that HY at 35 µM could dramatically suppress NF-κB nuclear translocation upon LPS stimulation (Figure 5C,D and Figure S2). These results facilitated us to identify that HY exerts its action to reverse LPS-induced inflammation, at least in part through the inhibition of NF-κB activation.

**Figure 6:**
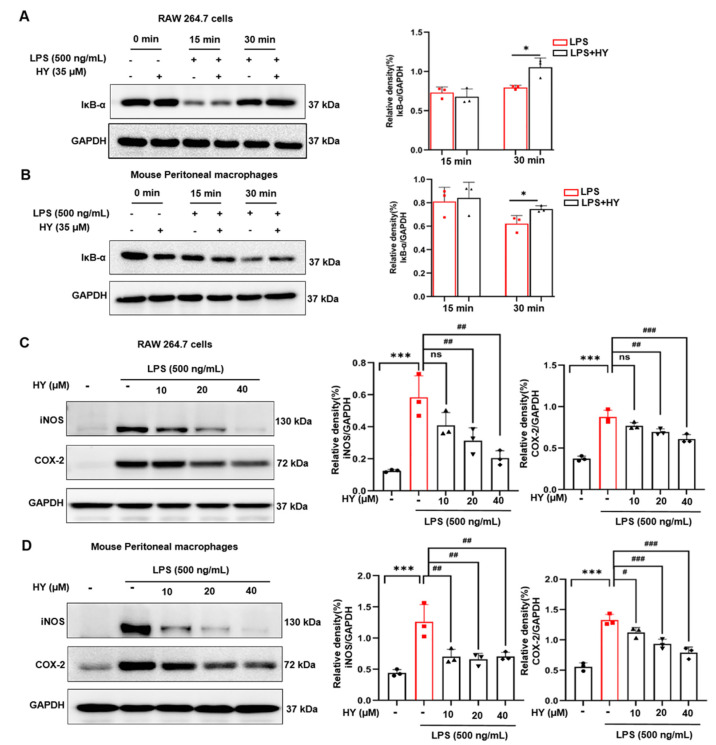
(**A**) Western blot analysis for the inhibitory effect of hyssopuside (HY) on the degradation and quantitative of inhibitor of NF-κB (IκB) in RAW 267.4 cells. (**B**) Western blot analysis for the inhibitory effect of HY on the degradation and quantitative of IκB in mouse peritoneal macrophages. Data are representative of three replicates and shown as mean ± SD. * *p* < 0.05 versus the lipopolysaccharide (LPS) group. (**C**) Western blot analysis of the inhibitory effects of HY on the protein expression level of inducible nitric oxide synthase (iNOS) and cyclooxygenase-2 (COX-2), and quantitative analysis of iNOS and COX-2 expression level in RAW 267.4 cells. (**D**) Western blot analysis of the inhibitory effects of HY on the protein expression level of iNOS and COX-2 and quantitative analysis of iNOS and COX-2 expression levels in mouse peritoneal macrophages. Glyceraldehyde 3-phosphate dehydrogenase (GAPDH) was detected and used as an internal control. Data are representative of three replicates and shown as mean ± SD; *** *p* < 0.05 versus the control group; # *p* < 0.05, ## *p* < 0.01, ### *p* < 0.001 versus the LPS-treated group.

We also utilized a Western blot study to verify whether HY suppresses NF-κB nuclear activation by inhibiting the downregulation of IκB upon LPS induction. In line with the forecast, the results (Figure 6A,B, Figures S3 and S4) clearly demonstrated that pre-treating cells for 3 h with HY could significantly inhibit LPS-induced degradation of IκB at 30 min, and then p65 translocated into the nucleus under the stimulation of LPS for 60 min. Furthermore, two major inflammatory enzymes, downstream to the NF-κB signaling, namely, iNOS and COX-2, were detected to be significantly reduced when HY was co-incubated in cells triggered with LPS for 24 h (Figure 6C,D, Figures S5 and S6). These data confirmed that HY could regulate LPS-induced production of NO, cytokines, and enzymes via inhibiting IκB degradation and thus suppressing nuclear translocation of NF-κB.

### 2.5. Effects of HY on MAPK Signaling Activation in LPS-Induced Macrophages

**Figure 7:**
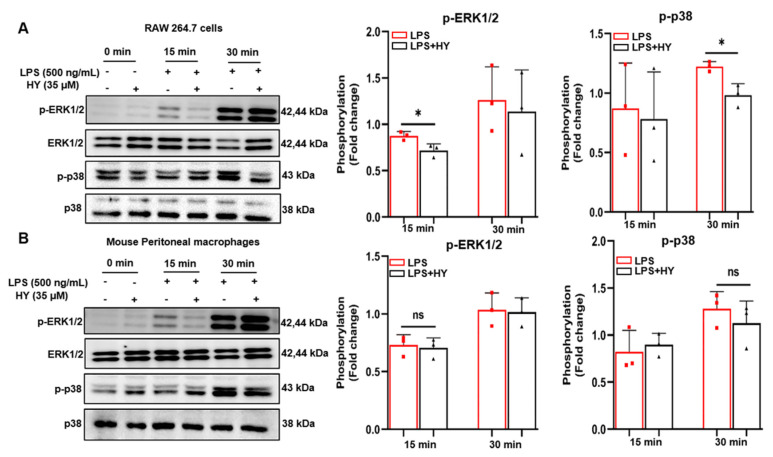
(**A**) Western blot analysis for the inhibitory effect of hyssopuside (HY) on the phosphorylation of mitogen-activated protein kinases (MAPKs), including extracellular signal-regulated kinase 1/2 (ERK1/2) and p-38 in LPS-induced RAW 267.4 cells treated with 35 μM HY for 15 or 30 min and quantitative of phosphorylation ERK1/2 and p-38. (**B**) Western blot analysis for the inhibitory effect of HY on the phosphorylation of MAPKs, including ERK1/2 and p-38 in LPS-induced mouse peritoneal macrophages treated with HY μM 35 for 15 or 30 min and quantitative of phosphorylation ERK1/2 and p-38. Data are representative of three replicates and shown as mean ± SD. * *p* < 0.05 versus the LPS group, ns, no significance.

It is known that in response to LPS induction, the activation of Toll-like receptor 4 (TLR4) leads to recruitment of the adaptor proteins such as myeloid differentiation protein 88 (MyD88) and TIR domain-containing adaptor protein inducing IFN (TRIF), followed by activation of extracellular signal-regulated kinase 1/2 (ERK1/2), p38 MAPK pathways, and NF-κB pathway [16], resulting in the production of inflammatory cytokines. Therefore, we sought to identify the possible mechanism of actions of HY in reducing the production of inflammatory cytokines. The signal transduction pathway of MAPK was determined in macrophages administrated with HY and induced with LPS. As expected, we found that HY moderately inhibited LPS-induced ERK (15 min) and p38 MAPK (30 min) (Figure 7A and Figures S7–S9) in RAW 246.7 cells, but HY slightly suppressed the protein level with no significance in primary peritoneal macrophages (Figure 7B and Figures S10–S12) since longer incubation time may be required for HY and macrophages.

### 2.6. HY interacts with COX-2 to Activate NF-κB

**Figure 8:**
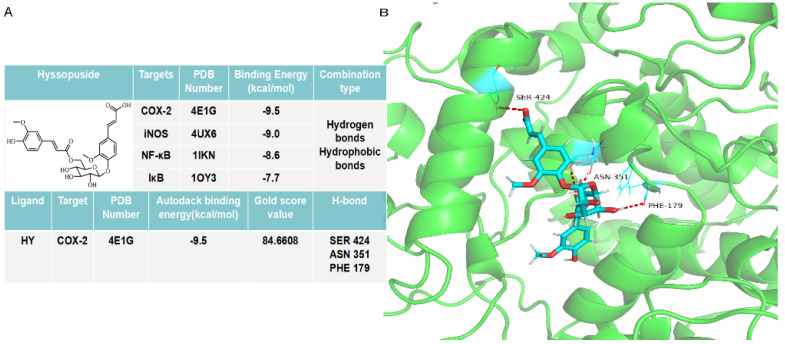
(**A**) The binding energy profile of molecular docking of hyssopuside (HY) with cyclooxygenase-2 (COX-2), inducible nitric oxide synthase (iNOS), nuclear factor kappa-B (NF-κB), and IκB (inhibitor of NF-κB). (**B**) The interaction between HY (in blue) and adjacent amino acid residues (in cyan) in COX-2 domain through hydrogen bonds (red dash).

The identification of a druggable target is important in the process of seeking out therapy drug molecules [17]. The results of molecular docking showed that the studied ligand, HY, possesses good to moderate affinity toward all the target proteins and enzymes used in this study with binding affinities ranging from −7.7 to −9.5 kcal/mol (Figure 8A). The best result was seen when HY was docked with COX-2 (−9.5 kcal/mol) enzyme with a considerable gold score 84.66. In addition to this enzyme, HY also efficiently interacted with iNOS (−9.0 kcal/mol) and NF-κB (−8.6 kcal/mol). These four bio-macromolecules are key events causing inflammatory conditions, and binding of HY with these can verify its anti-inflammatory effects. Interactions between HY and amino acid residues around the binding sites in COX-2 showed that (Figure 8B) HY could enter the binding cavity of COX-2 to form the complex through the hydrogen bonds with residues including Phe179, Asn351, and Ser424.

## 3. Discussion

Several species of the genus *Hyssopus* are used in folk medicine as anti-inflammatory, anti-asthmatic, and other medicinal treatments [18,19,20,21,22]. One of the species of this genus is *H. cuspidatus*, which is widely disseminated in the eastern Mediterranean, Central Asia, and southern Europe. Previous research showed that extract from *H. cuspidatus* rich in polyphenols effectively reduced the levels of pro-inflammatory mediators in vitro and in vivo [13]. However, there is little in the literature regarding the corresponding individual bioactive compound isolated from *H. cuspidatus* and its possible anti-inflammatory mechanism and action. In our previous phytochemical study, HY was found to inhibit NO production in RAW 264.7 cells stimulated with LPS [15]. The current research was conducted to provided significant insight into the underlying mechanisms of HY modulating the expression and production of pro-inflammatory mediators NO, iNOS, COX-2, IL-6, IL-1β, and TNF-α in LPS-stimulated RAW 267.4 cells and mouse peritoneal macrophages.

Although inflammation is a natural host defense system, its progressive production can cause organ dysfunctions to patients [23]. The most well-concerned inducer is LPS, the outer membrane of Gram-negative bacteria, which triggers TLR4 (a member of pattern recognition receptors) [24]. Upon LPS stimulation, serum IL-6, IL-1β, and TNF-α spring up and even exacerbate inflammation in patients [25]. Combined inhibition of IL-6, IL-1β, and TNF-α can alleviate multiple organ paralysis and improve host survival [26,27,28]. Here, we accumulated interesting evidence indicating that HY could suppress induction of iNOS and COX-2 (Figure 2F,G and Figure 6C,D) through the down-regulation of their promoter activities (Figure 5C,D) and subsequent production of NO (Figure 2D,E) in response to LPS stimulation.

An inflammatory disorder is identified as the production of excessive free radicals, reactive nitrogen species, and cytokines [29]. TNF-α is a vital cytokine in the inflammatory cytokine network that plays a part in initiating the release of IL-6 and IL-1β. As an endogenous pyrogen, TNF-α can cause fever and induce endotheliocytes and leukocytes to release a series of inflammatory mediators (NO, oxyradical, etc.) that may further promote TNF-α production [30]. IL-6 is one of the main cytokines in the expression of persistence and exacerbation inflammatory and autoimmune diseases such as rheumatoid arthritis, systemic sclerosis, asthma, and other diseases [31,32]. Therefore, reduction of the pro-inflammatory cytokines may be a part of a therapeutic treatment for preventing or treating inflammatory-related diseases. Our results showed that HY promoted the diminished of the overexpression of IL-6 and TNF-α (Figure 3A–F), showing that HY via transcriptional inhibition of IL-6 and TNF-α exerted its anti-inflammatory action.

MAPK signaling pathway has a certain bearing on the activation of NF-κB, at least or in part through the phosphorylation of IκB (an inhibitory protein of NF-κB), and plays a critical role in the translational regulation of pro-inflammatory cytokine synthesis [33]. Consequently, the observations of how HY inhibited these signal molecular transduction pathways were an adequate explanation for how HY suppresses NF-κB activation. Under basal conditions, NF-κB exists in the cytosol in a quiescent form bound to IκB. Upon being triggered by LPS, IκB is phosphorylated and undergoes proteolytic degradation, leading to the activated NF-κB translocating to the nucleus and activating its target genes, which include iNOS, COX-2, and pro-inflammatory cytokines, by binding to its consensus sequence in their promoter regions [16]. Results from Western blot analysis indicated that HY downregulates the degradation of IκB after LPS stimulation. Consistently, the immunofluorescence results verified that HY could suppress the translocation of NF-κB to the nucleus under LPS stimulation. Altogether, these results suggest that the inhibition of IκB and NF-κB activation by HY may account for the reduction of the LPS-induced pro-inflammatory cytokines. Besides important cytokines including IL-6 and TNF-α, NF-κB is well-identified play a critical role in the expression of inflammatory enzymes such as iNOS and COX-2. Therefore, we detected the level of protein expression of iNOS and COX-2. As expected, the results revealed that the expression of these two inflammatory enzymes was significantly diminished in cells treated with HY. The observation of iNOS being downregulated by the presence of HY may offer a rational explanation for the reduction of NO in the supernatants of the treated cells. Additionally, the ability of HY to reduce the LPS-induced activation of ERK1/2 and p38 may also contribute to a decrease in iNOS and COX-2. These results were in keeping with the fact that MAPKs are involved in regulating iNOS and COX-2 genes, since inhibiting the activity of MAPKs led to suppression of iNOS and COX-2 gene expression [34].

With the aim of developing molecules for anti-inflammation treatment, it is a core concern to identify the druggable target of HY [17,35]. Therefore, we further investigated the crucial target of HY with molecular modeling. As expected, the data further revealed that HY possesses good affinity with the key events causing inflammatory conditions (Figure 8A,B), making it a prospective drug-like molecule for clinical usage. Nevertheless, it is vital that elucidating the target of HY with more experiments than simply calculation, such as capillary electrophoresis and co-immunoprecipitation, may provide more evidence than molecular docking, which is important for identification of the direct target of HY and further utilization of HY in clinical practice.

In conclusion, our results demonstrated that HY treatment results in a decrease of iNOS, COX-2, and pro-inflammatory mediators by inhibiting the activation of NF-κB following LPS stimulation in RAW 264.7 cells and mouse peritoneal macrophages. Furthermore, our findings provide the first molecular basis for the anti-inflammatory properties of HY. Although HY was not used in physiological conditions in our in vitro model, our results open the window to inspire further pharmacological applications of this phenolic compound.

## 4. Materials and Methods

### 4.1. Plant Material and Prepared HY

HY was isolated from the aerial parts of *H. cuspidatus*, as per our previous description [15], and was kept in an air-tight and light-protected container until used. The purity was determined by HPLC (purity: ≥97%).

In the test, 10 mg HY was dissolved in 500 μL of dimethyl sulfoxide (DMSO) (Sigma-Aldrich, St. Louis, MO, USA) to make a 20 mg/mL stock solution. The HY stock was pre-diluted in medium prior to each treatment. In addition, the final concentration of DMSO was preserved below 0.1%.

### 4.2. Animals

Pathogen-free female C57BL/6 mice (20–22 g, 8–10 weeks) were obtained from the Experimental Animal Center, Jilin University (Changchun, Jilin, China). Mice were housed in a temperature- and humidity-controlled environment with a 12 h/12 h light/dark cycle. Animal experiments were performed with protocols approved by the Ethics Committee of Northeast Normal University (approval no: NENU/IACUC, AP20191225) and were conducted in accordance with the ARRIVE guidelines and the National Institutes of Health guide for the care and use of laboratory animals (NIH publication no. 8023, revised 1978).

### 4.3. Cell Culture and Treatment

The mouse RAW 264.7 macrophages (ATCC^®^ TIB-71^TM^) were acquired from ATCC (ATCC, Manasssas, VA, USA). The cells were cultured in complete medium, which was Dulbecco’s modified Eagle’s medium (DMEM) (Gibco, Thermo Fisher Scientific, Inc., Waltham, MA, USA) with antibiotics (100 U/mL penicillin and 100 µg/mL streptomycin) (Gibco, Thermo Fisher Scientific, Inc., MA, USA) and 10% fetal bovine serum (Gibco, Thermo Fisher Scientific, Inc., Waltham, MA, USA), maintained in a humidified incubator with 5% CO_2_ at 37 °C. The cells were sub-cultured when they reached 80% confluence and were used in all experiments from 3 to 7 generations.

### 4.4. Preparation and Culturing Primary Peritoneal Macrophages (PPMS)

Primary peritoneal macrophages of mice were extracted as previously described [6]. Briefly, mouse peritoneal macrophages were acquired from ascites of C57BL/6 mice, which were injected intraperitoneally (i.p.) with 1 mL 6% soluble starch for three consecutive days before sacrifice. Then, we harvested the macrophages and centrifuged them at 800 rpm for 5 min. The cell pellet was washed with 10 mL pre-cooling PBS twice and cultured in complete medium for 4 h until the medium was replaced for subsequent experiments.

### 4.5. Cell Viability Analysis

To detect the effect of HY on the viability of macrophages, 3-(4,5-dimetlthiazol-2-yl)-2,5-diphenyltetrazolium bromide (MTT) (Sigma-Aldrich, St. Louis, MO, USA) assay was carried out according to a previous study [36]. RAW 264.7 cells and primary peritoneal macrophages were seeded in 96-well plates 2 × 10^4^ cells/well). Cells were treated with HY at concentrations ranging from 10 to 80 μM for 24 h, and then cells were exposed to the addition of 20 μL of MTT solution (5 mg/mL in PBS) for 4 h at 37 °C with 5% CO_2_. After we discarded the culture supernatants, the formazan crystals were resolved with 100 μL DMSO. The absorbance at 490 nm was then determined using a plate reader (Molecular Devices, San Jose, CA, USA). The optical density of the untreated cells was taken as 100%.

### 4.6. NO Production Assay

As an indicator of NO production, the nitrite concentrations were detected by Griess’ reagent (Beyotime, Shanghai, China). Briefly, RAW 264.7 cells and primary peritoneal macrophages were seeded into 96-well plates (2 × 10^4^ cells per well) for 16 h and starved into the medium containing 1% FBS for 6 h. Cells were then treated with HY (35 μM) for 3 h in the presence of 500 ng/mL LPS for 24 h. The supernatants of the medium were collected for NO analysis. Equal volumes of supernatants and Griess’ reagent were mixed for 8 min. The nitrite ions formed a pink diazo dye by diazonium coupling reaction with N-(1-naphthyl) ethylenediamine. Finally, the absorbance was measured using a plate reader (Molecular Devices, San Jose, CA, USA) at 540 nm and compared with a standard nitrite curve ranging from 0 to 100 µM.

### 4.7. RNA Isolation, cDNA Synthesis, and Real-Time Quantitative Polymerase Chain Reaction (RT-qPCR)

To reveal the expression of inflammatory cytokines in macrophages, we inoculated RAW 264.7 cells and primary peritoneal macrophages in 12-well plates (5 × 10^5^ cells per well) for 16 h and starved in the medium containing 1% FBS for 6 h. Cells were then treated with HY (35 μM) for 3 h, and then in the presence of 500 ng/mL LPS for 12 h. The total RNA of cells was extracted with TRIzol reagent (Invitrogen, Carlsbad, CA, USA) and kept at −80 °C until use. The RNA samples were inverse transcribed cDNA using a TransScript SuperMix (TransGen Biotech, Beijing, China) according to the supplier’s protocols. Real-time PCR carried out using a FastStart Universal SYBR Green Master (Roche, Mannheim, Germany) with a Bio-Rad real-time PCR detection system. The PCR primers for specific genes were as follows: (mouse)

*Tnfα*: 5′-ACGTCGTAGCAAACCACCAA-3′ (sense) and 5′-GCAGCCTTGTCCCTTGAAGA-3′ (antisense); *Il6*: 5′-GAGACTTCCATCCAGTTGCCT-3′ (sense) and 5′-CAGGTCTGTTGGGAGT GGTA-3′ (antisense); *Il1b*: 5′-AACCTTTGACCTGGGCTGTC-3′ (sense) and 5′-ACGGGAAA GACACAGGTAGC-3′ (antisense); *Cox-2*: 5′-ATGACTGCCCAACTCCCATG-3′ (sense) and 5′-ACTGATGGGTGAAGTGCTGG-3′ (antisense); *Nos2*: 5′-CTATGGCCGCTTTGATGTGC-3′ (sense) and 5′-CACCCACCTCCAGTAGCATG-3′ (antisense); *Gapdh*: 5′-AGATCCCTCCAAAA TCAAGTGG-3′ (sense) and 5′-GGCAGAGATGATGACCCTTTT-3′ (antisense).

### 4.8. Enzyme-Linked Immunosorbent Assay (ELISA)

To reveal the secretion of inflammatory cytokines in macrophages, we determined the major constituents of extracellular matrix including IL-6, IL-1β, and TNF-α using the commercially available ELISA assay kits (Shanghai mlbio, Shanghai, China). RAW 264.7 cells and primary peritoneal macrophages were treated or untreated with HY (35 μM) for 3 h, and then in the presence of 500 ng/mL LPS for 24 h. The supernatants of the cells were harvested and measured by the supplier’s instructions, and the aabsorbance was monitored on a plate reader (Molecular Devices, San Jose, CA, USA) at 450 nm. The levels of IL-6, IL-1β, and TNF-α were derived from the absorbance in comparison to that of the control group.

### 4.9. Total and Nuclear Protein Extraction and Western Blot Analysis

We investigated the effects of HY on inhibiting LPS-induced expression and phosphorylation of importance proteins in NF-κB and MAPK signaling pathways. RAW 264.7 cells and primary peritoneal macrophages were treated with HY (35 μM) for 3 h and stimulated with 500 ng/mL of LPS at appropriate time points before harvesting. Total and nuclear proteins were extracted as previously reported [37]. In brief, for total proteins, the cells were harvested with PBS by centrifugation at 5000 rpm for 5 min and re-suspended to 0.33 g/mL of cell pellet in lysis buffer. After being lysed on ice for 10 min, the cell debris was then centrifuged at 12,000 rpm for 5 min. The clarified cell lysate was collected as total proteins after quantification using BCA protein assay kit. Then, it was heated at 100 °C for 10 min. For nuclear proteins, the cells were exposed to 100 μL cytoplasmic lysate buffer. After votexing for 5 s and incubation on ice for 6 min, repeated seven times, centrifugation was implemented at 12,000 rpm and 4 °C for 5 min. The cell lysates were harvested as cytoplasmic proteins, heated at 100 °C for 10 min, and stored at −80 °C until use. Then, the cell pallet was washed with 500 μL PBS and lysed with 50 μL nuclear protein extraction solution on ice for 1 h. Meanwhile, vortex was performed for 10 s every 5 min. After centrifugation, the supernatant was collected as nuclear proteins for further analysis after heating at 100 °C for 10 min.

The above proteins extracted were resolved on 12% sodium dodecyl sulfate polyacrylamide gel electrophoresis (SDS-PAGE) and transferred to polyvinylidene fluoride (PVDF) membranes (GE Healthcare Life Science, Marlborough, MA, USA). After being blocked with 5% skim milk at room temperature for 1.5 h, the membranes were incubated with specific primary antibodies including a dilution of a rabbit anti-IκB (1:1000) (catalog number 4812), a phosphospecific rabbit anti-p38 MAPK (1:1000) (catalog number 4511), a rabbit anti-ERK (1:1000) (catalog number 4605), a phosphospecific rabbit anti-ERK1/2 (1:1000) (catalog number 4370) (Cell Signaling Technology, Danvers, MA, USA), a mouse anti-p38 MAPK (1:500) (catalog number SC-7972), a goat anti-COX2 antibody (1:500) (catalog number SC-1745) (Santa Cruz, CA, USA), a rabbit anti-iNOS antibody (1:1000) (catalog number18985-1-AP), a rabbit anti-NF-κB p65 (1:1000) (catalog number10745-1-AP), a rabbit anti-Histone-H3 (1:1000) (catalog number 17168-1-AP), and a mouse anti-GDPAH (1:1000) (catalog number 60004-1-Ig) (Proteintech Group, Inc., Wuhan, China) at 4 °C for 16 h. After being rinsed with buffer solution containing 0.1% Tween-20 for 10 min for three times, the membranes were incubated with horseradish peroxidase-conjugated secondary antibody, an HRP-conjugated goat anti-mouse IgG (H+L) (catalog number SA00001-1) (1:5000), an HRP-conjugated goat anti-rabbit IgG (H+L) (catalog number SA00001-2) (1:5000), and an HRP-conjugated rabbit anti-goat IgG (H+L) (catalog number SA00001-4) (1:5000) at room temperature for 1.5 h. The immunoreactive bands were visualized using ECL substrate (Beyotime, Shanghai, China), and densitometric analysis was performed using ImageJ software (exploit at the National Institutes of Health, USA, http://rsb.info.nih.gov/ij, accessed on 25 October 2020).

### 4.10. Immunofluorescence Assay

Immunofluorescence was performed visualize NF-κB nuclear localization upon LPS stimulation. RAW 264.7 cells and primary peritoneal macrophages were seeded in 96-well (5 × 103 cells per well) fluorescent plates left treated or untreated with HY (35 μM) for 3 h. Cells were then stimulated with 500 ng/mL of LPS for 60 min. After treatment, cells were fixed with 4% paraformaldehyde (Sigma-Aldrich, Saint Louis, MO, USA) dissolved in PBS for 20 min at RT. Cells were then washed three times with PBS for 3 min each time. Next, cells were permeabilized with 0.2% Triton X-100 in PBS for 10 min. The cells were washed three times with PBS then blocked with 5% BSA in PBS for 30 min. Cells were incubated with a primary antibody rabbit anti-NF-κB antibody staining (1:200) (antibody catalog number 10745-1-AP, Proteintech Group, Inc., Wuhan, China) overnight at 4 °C. After being washed three times, cells were incubated with secondary antibodies at a 1:500 dilution of Alexa488-conjugated anti-rabbit IgG (Proteintech Group, Inc., Wuhan, China) plus 1 µg/mL of DAPI (Sigma-Aldrich, Saint Louis, MO, USA) (for nuclear staining) for 5 min in the dark at RT. Cells were washed four times with PBS (5 min each). Finally, cells were observed on a BD Pathway™ Bioimager (BD Biosciences, Franklin Lakes, NJ, USA) with 100× magnification.

### 4.11. Molecular Modeling

To elucidate the detailed interaction between HY and NF-κB pathway, we performed molecular docking using Autodock 1.5.6 software and Gold 5.2 software according to previously published methods [38,39]. In brief, ligand molecule HY was constructed by the ChemBio3D Ultra 14.0 software using mm2 to optimize the structure to energy minimization to create a sdf file for later use. The co-crystal 3D structures of target proteins NF-κB (PDB code: 1IKN), IκB (PDB code: 1OY3), iNOS (PDB code: 4UX6), and COX-2 (PDB code: 4E1G) were downloaded from the RSCB Protein Data Bank (https://www.rcsb.org/ accessed on 25 January 2020) and saved as pdb format. The primary ligand of proteins was extracted, and water molecules in the structure were removed. After the addition of drogen atoms, the protomol file was generated to establish the docking envelope, and dock program with default parameters was operated for docking calculations. The Autodock 1.5.6 software was used to calculate the binding energy between proteins and HY. The Gold 5.2 software was used to further calculate the gold score. The docking diagrams were visualized by PyMol.

### 4.12. Statistical Analysis

All data were presented as mean ± SD according to at least three independent experiments and then analyzed by Graphpad Prism 9 software (GraphPad Software, San Diego, CA, USA). One-way ANOVA was used to make statistical comparison, with Tukey–Kramer multiple comparisons. In all analyses, data were justified by means of Shapiro–Wilk normality test, Kolmogorov–Smirnov test (with Dallal–Wilkinson–Lilliefor *p*-value), and D’Agostino and Pearson omnibus normality test for presentation as an average and standard deviation in the normal distribution of features, with a *p*-value (*p* < 0.05) being regarded as statistically significant.

## Data Availability

Not applicable.

## Supplementary Materials

The following are available online at https://www.mdpi.com/article/10.3390/ijms222212128/s1.

## Abbreviations

COX-2	Cyclooxygenase-2
DMEM	Dulbecco’s modified Eagle’s medium
DAPI	4′,6-Diamidino-2-phenylindole4′,6-diamidino-2-phenylindole
ELISA	Enzyme-linked immunosorbent assay
ERK1/2	Extracellular signal-regulated kinase 1/2
FBS	Fetal bovine serum
GAPDH	Glyceraldehyde 3-phosphate dehydrogenase
HY	Hyssopuside
IκB	Inhibitor of NF-κB
IL-6	Interleukin-6
IL-1β	Interleukin-1β
iNOS	Inducible nitric oxide synthase
LPS	Lipopolysaccharide
MAPK	Mitogen-activated protein kinase
MTT	3-(4,5-Dimethylthiazol-2yl)-2,5-diphenyltetrazolium bromide
NF-κB/p65	Nuclear factor kappa-B
NO	Nitric oxide
RT-qPCR	Real-time quantitative polymerase chain reaction
TNF-α	Tumor necrosis factor-α
