# Supplementary material for: Discovery of Phenolic Glycoside from Hyssopus cuspidatus Attenuates LPS-Induced Inflammatory Responses by Inhibition of iNOS and COX-2 Expression through Suppression of NF-κB Activation
Source: Int J Mol Sci. 2021 Nov 9;22(22):12128. doi: 10.3390/ijms222212128 (PMC8623068; doi:10.3390/ijms222212128)
Supplement: Supplementary file 1 [file ijms-22-12128-s001.zip › ijms-1455080-supplementary.pdf]

Supplementary Materials for

Discovery of Phenolic Glycoside from *Hyssopus cuspidatus* Attenuates LPS-Induced Inflammatory Responses by Inhibition of iNOS and COX-2 Expression through Suppression of NF-κB Activation

Xingyu Liu[1], Jie Su[1], Geng Wang[2], Lihua Zheng[2], Guannan Wang[1], Ying Sun[1], Yongli Bao[1], Shuyue Wang[1,*] and Yanxin Huang[2,*]

[1] National Engineering Laboratory for Druggable Gene and Protein Screening, Northeast Normal University, Changchun 130117, China; liuxy013@nenu.edu.cn (X.L.); suj445@nenu.edu.cn (J.S.); wanggn258@nenu.edu.cn (G.W.); suny040@nenu.edu.cn (Y.S.); baoyl800@nenu.edu.cn (Y.B.)

[2] NMPA Key Laboratory for Quality Control of Cell and Gene Therapy Medicine Products, Northeast Normal University, Changchun 130117, China; wangg666@nenu.edu.cn (G.W.); zhenglh015@nenu.edu.cn (L.Z.)

* Correspondence: wangsy171@nenu.edu.cn (S.W.); huangyx356@nenu.edu.cn (Y.H.); Tel.: +86-431-8916-5922 (S.W. & Y.H.); Fax: +86-431-8916-5917 (S.W. & Y.H.)

Compound	COX-2 (kcal/mol)	iNOS (kcal/mol)	NF-κB (kcal/mol)	IκB (kcal/mol)
2′-acetylacteoside	-9.2	-7.8	-8.3	-8.5
Echinacoside	-8.7	-7.9	-10.1	-8.6
Gastrodin	-7.6	-6.7	-7.1	-6.4
Isoacteoside	-9.0	-9.6	-8.2	-9.2
Salidroside	-7.2	-8.1	-6.8	-6.4
Verbascoside	-10.2	-10	-8.4	-9.2
Hyssopuside	-9.5	-9.0	-8.6	-7.7

Figure S1: The binding energy profile of molecular docking of natural phenolic glycosides with COX-2, iNOS, NF-κB and IκB.

Figure 5C: p65

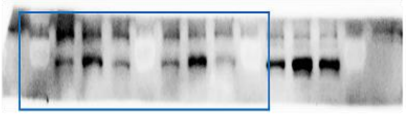

Lane Description:

p65 in RAW 264.7 cells

Lane 1: marker for 70 KDa

Lane 2: Control

Lane 3: Treated with LPS for 60 min

Lane 4: Treat with LPS and 35 μM HY for 60 min

p65 in primary peritoneal macrophages

Lane 5: marker for 70 KDa

Lane 6: Control

Lane 7: Treated with LPS for 60 min

Lane 8: Treat with LPS and 35 μM HY for 60 min

Lane 9: marker for 70 KDa

Lane 10-12: Not appared in the manuscript

Figure 5C: Histone-H3

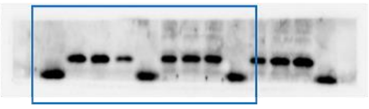

Lane Description:

Histone-H3 in RAW 264.7 cells

Lane 1: marker for 15 KDa

Lane 2: Control

Lane 3: Treated with LPS for 60 min

Lane 4: Treat with LPS and 35 μM HY for 60 min

Histone-H3 in primary peritoneal macrophages

Lane 5: marker for 15 KDa

Lane 6: Control

Lane 7: Treated with LPS for 60 min

Lane 8: Treat with LPS and 35 μM HY for 60 min

Lane 9: marker for 15 KDa

Lane 10-12: Not appared in the manuscript

Figure 5C: GAPDH

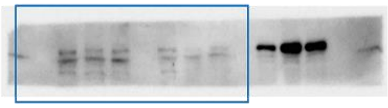

Lane Description:

GAPDH in RAW 264.7 cells

Lane 1: marker for 35 KDa

Lane 2: Control

Lane 3: Treated with LPS for 60 min

Lane 4: Treat with LPS and 35 μM HY for 60 min

GAPDH in primary peritoneal macrophages

Lane 5: marker for 35 KDa

Lane 6: Control

Lane 7: Treated with LPS for 60 min

Lane 8: Treat with LPS and 35 μM HY for 60 min

Lane 9: marker for 35 KDa

Lane 10-12: Not appared in the manuscript

Figure S2: The whole un-cropped western blots images of figure 5C and 5D.

Figure 6A: IκBα

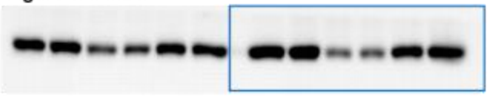

Lane Description

Lane 1-6: Not appared in the manuscript.

IκBα in RAW 264.7 cells

Lane 7: marker for 35 KDa

Lane 8: Control

Lane 9: Treated with HY for 3 h

Lane 10: Treat with LPS for 15 min

Lane 11: Treat with LPS and 35 μM HY for 15 min

Lane 12: Treat with LPS for 30 min

Lane 13: Treat with LPS and 35 μM HY for 30 min

Figure 6A: GAPDH

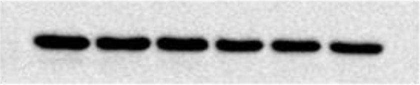

Lane Description

IκBα in RAW 264.7 cells

Lane 1: marker for 35 KDa

Lane 2: Control

Lane 3: Treated with HY for 3 h

Lane 4: Treat with LPS for 15 min

Lane 5: Treat with LPS and 35 μM HY for 15 min

Lane 6: Treat with LPS for 30 min

Lane 7: Treat with LPS and 35 μM HY for 30 min

Figure S3: The whole un-cropped western blots images of figure 6A.

Figure 6B: IκBα

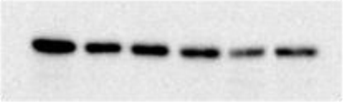

Lane Description

IκBα in primary peritoneal macrophages

Lane 1: marker for 35 KDa

Lane 2: Control

Lane 3: Treated with HY for 3 h

Lane 4: Treat with LPS for 15 min

Lane 5: Treat with LPS and 35 μM HY for 15 min

Lane 6: Treat with LPS for 30 min

Figure 6B: GAPDH

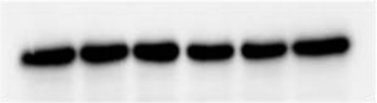

Lane Description

IκBα in primary peritoneal macrophages

Lane 1: marker for 35 KDa

Lane 2: Control

Lane 3: Treated with HY for 3 h

Lane 4: Treat with LPS for 15 min

Lane 5: Treat with LPS and 35 μM HY for 15 min

Lane 6: Treat with LPS for 30 min

Figure S4: The whole un-cropped western blots images of figure 6B.

Figure 6C: iNOS

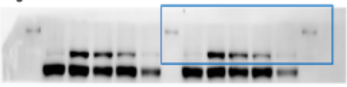

Lane Description:

Lane 1-6: Not appared in the manuscript.

iNOS in RAW 264.7 cells

Lane 7: marker for 150 KDa

Lane 8: Control

Lane 9: Treated with LPS

Lane 10: Treat with LPS and 10 μM HY for 24 h

Lane 11: Treat with LPS and 20 μM HY for 24 h

Lane 12: Treat with LPS and 40 μM HY for 24 h

Lane 13: marker for 150 KDa (top)

Figure 6C: COX-2

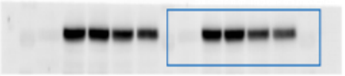

Lane Description:

Lane 1-6: Not appared in the manuscript.

COX-2 in RAW 264.7 cells

Lane 7: marker for 70 KDa

Lane 8: Control

Lane 9: Treated with LPS

Lane 10: Treat with LPS and 10 μM HY for 24 h

Lane 11: Treat with LPS and 20 μM HY for 24 h

Lane 12: Treat with LPS and 40 μM HY for 24 h

Lane 13: marker for 70 KDa

Figure 6C: GAPDH

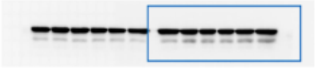

Lane Description:

Lane 1-6: Not appared in the manuscript.

GAPDH in RAW 264.7 cells

Lane 7: marker for 35 KDa

Lane 8: Control

Lane 9: Treated with LPS

Lane 10: Treat with LPS and 10 μM HY for 24 h

Lane 11: Treat with LPS and 20 μM HY for 24 h

Lane 12: Treat with LPS and 40 μM HY for 24 h

Lane 13: marker for 35 KDa

Figure S5: The whole un-cropped western blots images of figure 6C.

Figure 6D: iNOS

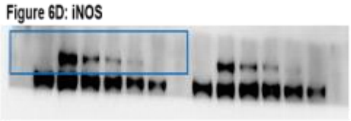

Lane Description:

iNOS in primary peritoneal macrophages

Lane 1: marker for 150 KDa

Lane 2: Control

Lane 3: Treated with LPS

Lane 4: Treat with LPS and 10 μM HY for 24 h

Lane 5: Treat with LPS and 20 μM HY for 24 h

Lane 6: Treat with LPS and 40 μM HY for 24 h

Lane 7: Treat with LPS and 80 μM HY for 24 h

Lane 8: marker for 150 KDa

Lane 9-16: Not appared in the manuscript.

Figure 6D: COX-2

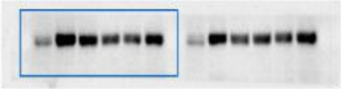

Lane Description:

COX-2 in primary peritoneal macrophages

Lane 1: marker for 150 KDa

Lane 2: Control

Lane 3: Treated with LPS

Lane 4: Treat with LPS and 10 μM HY for 24 h

Lane 5: Treat with LPS and 20 μM HY for 24 h

Lane 6: Treat with LPS and 40 μM HY for 24 h

Lane 7: Treat with LPS and 80 μM HY for 24 h

Lane 8: marker for 150 KDa

Lane 9-16: Not appared in the manuscript.

Figure 6D: GAPDH

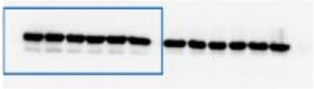

Lane Description:

iNOS in primary peritoneal macrophages

Lane 1: marker for 150 KDa

Lane 2: Control

Lane 3: Treated with LPS

Lane 4: Treat with LPS and 10 μM HY for 24 h

Lane 5: Treat with LPS and 20 μM HY for 24 h

Lane 6: Treat with LPS and 40 μM HY for 24 h

Lane 7: Treat with LPS and 80 μM HY for 24 h

Lane 8: marker for 150 KDa

Lane 9-16: Not appared in the manuscript.

Figure S6: The whole un-cropped western blots images of figure 6D.

Figure 7A: p-ERK

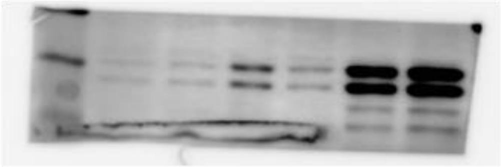

Lane Description:

p-ERK in RAW 264.7 cells

Lane 1: marker for 43 KDa

Lane 2: Control

Lane 3: Treated with 35 μM HY

Lane 4: Treat with LPS for 15 min

Lane 5: Treat with LPS and 35 μM HY for 15 min

Lane 6: Treat with LPS for 30 min

Lane 7: Treat with LPS and 35 μM HY for 30 min

Figure 7A: ERK

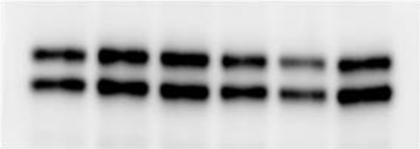

Lane Description:

ERK in RAW 264.7 cells

Lane 1: marker for 43 KDa

Lane 2: Control

Lane 3: Treated with 35 μM HY

Lane 4: Treat with LPS for 15 min

Lane 5: Treat with LPS and 35 μM HY for 15 min

Lane 6: Treat with LPS for 30 min

Lane 7: Treat with LPS and 35 μM HY for 30 min

Figure S7: The whole un-cropped western blots images of figure 7A.

Figure 7A: p-p38

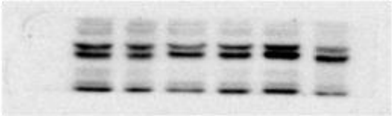

Lane Description:

p-p38 in RAW 264.7 cells

Lane 1: marker (too weak)

Lane 2: Control

Lane 3: Treated with 35 μM HY

Lane 4: Treat with LPS for 15 min

Lane 5: Treat with LPS and 35 μM HY for 15 min

Lane 6: Treat with LPS for 30 min

Lane 7: Treat with LPS and 35 μM HY for 30 min

Figure 7A: p38

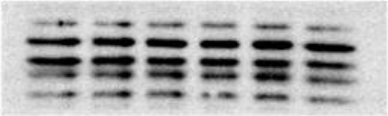

Lane Description:

p-p38 in RAW 264.7 cells

Lane 1: Control

Lane 2: Treated with 35 μM HY

Lane 3: Treat with LPS for 15 min

Lane 4: Treat with LPS and 35 μM HY for 15 min

Lane 5: Treat with LPS for 30 min

Lane 6: Treat with LPS and 35 μM HY for 30 min

Figure S8: The whole un-cropped western blots images of figure 7A.

Figure 7A: GAPDH

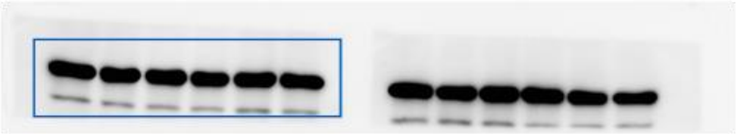

Lane Description:

GAPDH in RAW 264.7 cells

Lane 1: marker

Lane 2: Control

Lane 3: Treated with 35 μM HY

Lane 4: Treat with LPS for 15 min

Lane 5: Treat with LPS and 35 μM HY for 15 min

Lane 6: Treat with LPS for 30 min

Lane 7: Treat with LPS and 35 μM HY for 30 min

Lane 8: marker

Lane 9-14: Not appared in the manuscript

Figure S9: The whole un-cropped western blots images of GAPDH for figure 7A.

Figure 7B: p-ERK

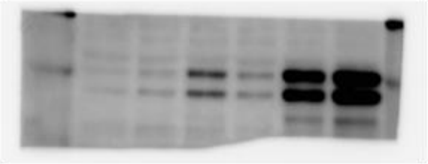

Lane Description:

p-ERK in primary peritoneal macrophages

Lane 1: marker for 43 KDa

Lane 2: Control

Lane 3: Treated with 35 μM HY

Lane 4: Treat with LPS for 15 min

Lane 5: Treat with LPS and 35 μM HY for 15 min

Lane 6: Treat with LPS for 30 min

Lane 7: Treat with LPS and 35 μM HY for 30 min

Figure 7B: ERK

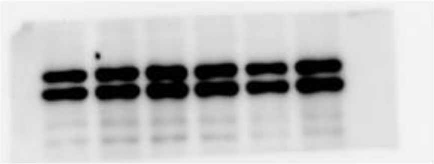

Lane Description:

ERK in primary peritoneal macrophages

Lane 1: Control

Lane 2: Treated with 35 μM HY

Lane 3: Treat with LPS for 15 min

Lane 4: Treat with LPS and 35 μM HY for 15 min

Lane 5: Treat with LPS for 30 min

Lane 6: Treat with LPS and 35 μM HY for 30 min

Lane 7: marker (too weak)

Figure S10: The whole un-cropped western blots images of figure 7B.

Figure 7B: p-p38

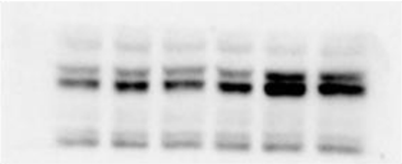

Lane Description:

p-p38 in primary peritoneal macrophages

Lane 1: marker (too weak)

Lane 2: Control

Lane 3: Treated with 35 μM HY

Lane 4: Treat with LPS for 15 min

Lane 5: Treat with LPS and 35 μM HY for 15 min

Lane 6: Treat with LPS for 30 min

Lane 7: Treat with LPS and 35 μM HY for 30 min

Figure 7B: p38

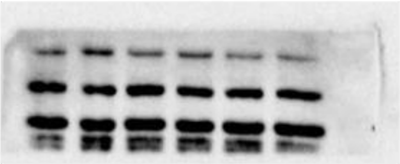

Lane Description:

p38 in primary peritoneal macrophages

Lane 1: Control

Lane 2: Treated with 35 μM HY

Lane 3: Treat with LPS for 15 min

Lane 4: Treat with LPS and 35 μM HY for 15 min

Lane 5: Treat with LPS for 30 min

Lane 6: Treat with LPS and 35 μM HY for 30 min

Lane 7: marker (too weak)

Figure S11: The whole un-cropped western blots images of figure 7B.

Figure 7B: GAPDH

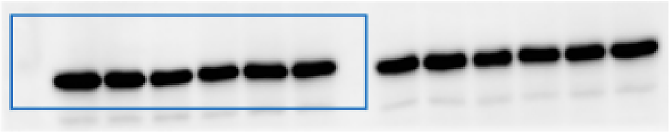

Lane Description:

GAPDH in primary peritoneal macrophages

Lane 1: marker for 35 KDa

Lane 2: Control

Lane 3: Treated with 35 μM HY

Lane 4: Treat with LPS for 15 min

Lane 5: Treat with LPS and 35 μM HY for 15 min

Lane 6: Treat with LPS for 30 min

Lane 7: Treat with LPS and 35 μM HY for 30 min

Lane 8: marker for 35 KDa

Lane 9-14: Not appared in the manuscript

Figure S12: The whole un-cropped western blots images of GAPDH for figure 7B.
